# Chlorpropamide in Parkinson's Disease

**Published:** 1964-01

**Authors:** P. C. Barrington

**Affiliations:** Medical Registrar, United Bristol Hospitals


### CHLORPROPAMIDE IN PARKINSON'S DISEASE

BY

P. C. BARRINGTON, M.B., M.R.C.P.

*Medical Registrar, United Bristol Hospitals*

Several authors have reported beneficial effects from the oral hypoglycaemic agent chlorpropamide in Parkinson's disease (Gillhespy and Paton, 1960; Robertson, 1961). These results are empirical, and so far no good theoretical reason for such effects has been put forward. The background to the use of antidiabetic drugs in Parkinson's disease includes the following observations, but their significance is not yet clear:

(1) There is evidence of a connection between abnormal glucose tolerance and post-encephalitic Parkinson's disease (Gillhespy and Paton, 1960; Terrana and Adragna, 1951). McCowan, Harris and Mann (1926) give glucose tolerance curves for a group of patients with post-encephalitic Parkinsonism which are clearly diabetic in type.

(2) Electrocoagulation in the basal ganglia in a diabetic with Parkinson's disease ameliorated both conditions (Gillingham, Watson, Donaldson and Naughton, 1960).

(3) In 1958 a diabetic with Parkinson's disease showed improvement of both conditions when started on Tolbutamide (Gates and Hyman, 1958), and these authors later found that cases of Parkinson's disease without diabetes also improved. A subsequent double-blind trial did not confirm this (Heller, DeJong and Magee, 1961).

(4) Dresel and Lewy (1921) described lesions in the globus pallidus in four diabetics who died in diabetic coma. Bodechtel and Erbsloh (1958) agree that changes do occur in the globus pallidus in diabetes, but suggest that they are not specific for diabetes, and are found elsewhere in the brain.

The present trial was not intended to throw any further light on the anomalies of glucose metabolism in Parkinson's disease, but was undertaken because there has so far been no well-controlled trial of chlorpropamide in this condition. Chlorpropamide is a drug which can cause hypoglycaemia both in diabetic and non-diabetic subjects which may be severe and prolonged if taken in too large a dose (Putelat, Bouhey, Lacroix and Veillet, 1962). This is a potential hazard to elderly arteriosclerotic patients, and is one reason why the drug should not be used in Parkinson's disease unless its value is clear.

### METHOD

The trial was double-blind. Eighteen volunteer patients from the neurological clinic were assigned at random to two groups, A and B. Each patient was seen five times at 3-week intervals, and at each of the first four visits a bottle containing 21 tablets was issued. The patient was told that each bottle might contain the actual drug or dummy tablets. In fact, group B received 100 mg. chlorpropamide daily for the first 6 weeks, while group A received the dummy. This was in addition to any previous medication, which was continued without change throughout the trial. For the final 6 weeks the groups were crossed over. At each visit the patient was assessed by one observer in three different ways:

(1) The patient was asked whether he had noticed any improvement or worsening since starting the last lot of tablets.

(2) The time taken to perform each of sixteen actions in a standard way was measured. These actions included tests of manual dexterity and others involving trunk and leg muscles, and were based on movements common in everyday life to minimise the effects of practice. The patients were asked each time to perform the test as rapidly as possible, and an attempt was made to keep the level of exhortation constant. The shorter tests were done twice at each visit.

*Tests*

*a.* Open and close a safety pin.

*b.* Cork and uncork a bottle.

*c.* Fold a letter and put it in an envelope.

*d.* Unscrew and screw up a jar top.

*e.* Open a matchbox and strike a match.

*f.* Slice a block of plasticine.

*g.* Transfer food from one plate to another using a fork.

*h.* Eat soup with a spoon.

*i.* Drink a glass of water.

*j.* Put on a dressing-gown.

*k.* Put on shoes.

*l.* Turn through 360 degrees.

*m.* Climb on and off a couch.

*n.* Sit down and stand again.

*o.* Walk a measured distance.

*p.* Mount a step ten times.

Test of writing and drawing were given on a standard sheet for each hand in turn. The sheets were coded with random numbers and shuffled. Later the five consecutive tests for each patient were arranged in order of excellence and scored 1 (best) to 5 (worst).

(3) Each limb was graded 0 (normal) to 5 (severely affected) for rigidity and tremor separately. No attempt was made to assess trunk muscles. Rigidity was assessed by passive movement at all joints of the limb, and the grade given was determined by the muscles most affected.

### RESULTS

1. *Subjective impression.* Each patient received the same treatment for two successive 3-week periods and was then crossed over. The periods have been shown separately in Table I, in case there is a delayed effect due to chlorpropamide. In fact, as many patients felt better on the dummy as on chlorpropamide, and there did not appear to be any delayed improvement due to the latter.

TABLE I

Patients' impressions of treatment with chlorpropamide or a dummy, for each 3 week period, both groups combined.

| | Chlorpropamide | | Dummy | |
|---|---|---|---|---|
| Period | 1 | 2 | 1 | 2 |
| Worse | 4 | 0 | 5 | 8 |
| Better | 3 | 4 | 6 | 1 |
| Same | 11 | 13 | 7 | 8 |

2. *Timed tests.* For individual patients at each visit a figure for the time taken to complete the tests was required. Since the time taken by the different tests differed considerably the greater influence upon the average time of tests with longer duration was eliminated by using the log transformation of the time. Each patient acted as his own control; therefore the antilog of the mean log time was expressed as a percentage of the mean of the five antilogs for his five visits.

The results for the two groups were then averaged separately to give the figures in Table II.

TABLE II

Time to complete the tests expressed as a percentage of the mean time (see text).

| Visit | 1 | 2 | 3 | 4 | 5 |
|---|---|---|---|---|---|
| | | | Chlorpropamide | | |
| A | 110 | 106 | 98 | 92 | 94 |
| | Chlorpropamide | | | | |
| B | 112 | 106 | 98 | 92 | 92 |

There is little difference in the results for group A and group B, but there is an inprovement of about 20 per cent from visit one to visit five, which is presumably due to the effects of practice. In group B only one subject showed a lower score during the chlorpropamide period than during the control period.

In case tests of unaffected limbs were diluting the effect of chlorpropamide, the results were analysed again and only counted if one of the limbs involved in the particular test had achieved a clinical score for rigidity of 2 on more than one occasion. It was found that rigidity correlated better with time to complete the tests than did tremor. The results were substantially the same.

It was found that in most cases draughtsmanship improved from visit one to visit five, so that the sheet for the latter usually scored 1. Averaging the scores for each group including left and right hand results together gave the results in Table III. Again the improvement due to chlorpropamide, if any, is less than that attributable to practice.

TABLE III

Tests of drawing and writing. The figures represent the average score at each visit, the possible range being from 1 to 5. There is a steady improvement from visit 1 to visit 5 in each group.

| Visit | 1 | 2 | 3 | 4 | 5 |
|---|---|---|---|---|---|
| | | | Chlorpropamide | | |
| A | 4·3 | 4·0 | 2·8 | 2·6 | 1·2 |
| | Chlorpropamide | | | | |
| B | 4·1 | 3·5 | 3·1 | 2·8 | 1·6 |

3. *The total scores* for eight patients in each group for rigidity and tremor are set out in Table IV for each of the last four visits (some of the patients were not tested at the first visit). There is a wide variation in the results during the control periods as might be expected with clinical assessment. However, there is no consistent pattern of improvement attributable to chlorpropamide. In particular, the improvement in tremor in group A is not reflected in group B.

TABLE IV

Total scores for rigidity and tremor for eight patients in each group at each of the last 4 visits.

| | Visit | 2 | 3 | 4 | 5 |
|---|---|---|---|---|---|
| | | | | Chlorpropamide | |
| A | Tremor | 38 | 43 | 30 | 30 |
| | Rigidity | 27 | 34 | 23 | 33 |
| | | Chlorpropamide | | | |
| B | Tremor | 44 | 39 | 41 | 39 |
| | Rigidity | 76 | 70 | 72 | 84 |

### DISCUSSION

It is difficult to devise tests which give useful information about the degree of limitation in Parkinson's disease, because it varies from time to time, particularly with emotion, and because rigidity, tremor and slowness also vary independently and affect different muscle groups in different patients. In this trial emotional factors were reduced by making it double-blind, and variation between patients was reduced by crossing over. The tests were based on those used at the Frenchay Hospital neurosurgical unit for assessing cases before and after operations on the basal ganglia, with the modification that time rather than subjective impression was used as the measure of performance when possible.

No benefit from chlorpropamide in the dose used (100 mg./day) was demonstrated. However, the results also suggest that after very few repetitions of complicated actions there may be a considerable improvement in performance due to practice, and it may be that tests using simpler actions such as some of those described by DeJong and Burns (1962) would have allowed smaller effects due to chlorpropamide to show up. Our results make it unlikely that such effects would be large enough to be clinically useful.

### SUMMARY

A double-blind crossover trial of chlorpropamide 100 mg./day in addition to their usual drugs in eighteen patients with Parkinson's disease is reported. Chlorpropamide did not prove better than the dummy as judged subjectively, by timed tests, or by its effect on rigidity and tremor.

*Acknowledgements*

I should like to thank Dr. G. Herdan for statistical advice and other help, the B.R.I. outpatient department nursing staff for practical assistance, and Dr. A. M. G. Campbell and Professor C. Bruce Perry for their encouragement. The chlorpropamide and dummy tablets were kindly supplied by Harvey Pharmaceuticals, Sandwich, Kent.

REFERENCES

Bodechtel, G., Erbsloh, F. (1958). Handbuch der speziellen Pathologischen Anatomieu. Histologie. XIII/2B 1720. Springer, Berlin.

DeJong, J. D., Burns, B. D. (1962). *Neurology* (Minneap.) **12**, 402.

Dresel, K., Lewy, F. H. (1921). *Berl. Klin. Woch.* 739.

Gates, E. W., Hyman, I. (1960). *J.A.M.A.*, **172**, 1351.

Gillhespy, R. O., Paton, A. (1960). *Brit. Med. J.*, **ii**, 1597.

Gillingham, F. J., Watson, W. S., Donaldson, A. A., Naughton, J. A. L. (1960). *Brit. Med. J.*, **ii**, 1401.

Heller, G. L., DeJong, R. N., Magee, K. R. (1961). *J.A.M.A.*, **176**, 148.

McCowan, P. K., Harris, J. S., Mann, S. A. (1926). *Lancet*, **i**, 802.

Putelat, R., Bouhey, J., Lacroix, M., Veillet, J. (1962). *J. Med. Lyon*, **43**, 265.

Robertson, J. (1961). *Brit. Med. J.*, **i**, 363,

Terrana, V., Adragna, S. (1951). *Rass. Neurol. Veg.*, **8**, 429.

